# A Thyrotoxic Periodic Paralysis Case Study: From Weakness to Wellness

**DOI:** 10.7759/cureus.47820

**Published:** 2023-10-27

**Authors:** Sharafath Hussain Zahir Hussain, Salam Al-Alousi, Lakshmi B Keshav, Aara Tabassum Zahir Hussain, Kirupananthan Seenithamby

**Affiliations:** 1 Medicine, University Hospitals of Leicester NHS Trust, Leicester, GBR; 2 Acute Medicine, University Hospitals of Leicester NHS Trust, Leicester, GBR; 3 Medicine, Nottingham University Hospitals NHS Trust, Nottingham, GBR; 4 Medicine and Surgery, Sri Ramachandra Institute of Higher Education and Research, Chennai, IND

**Keywords:** muscle weakness, proximal limb weakness, lower limb hyporeflexia, asian origin, hyperkalemic paralysis, hyperkalemic periodic paralysis, hyperthyroidism, thyrotoxic hypokalemic periodic paralysis

## Abstract

Hypokalaemic periodic paralysis (HPP) is a rare disorder characterized by episodic attacks of muscle weakness and hypokalaemia. Numerous factors contributing to HPP have been identified, encompassing both hereditary and familial origins as well as acquired factors. In this context, we highlight thyrotoxicosis causing HPP. We present a case of a 40-year-old Asian individual who presented with episodes of sudden onset bilateral proximal limb weakness and palpitations. Laboratory investigations revealed severe hypokalaemia (serum potassium: 1.8 mmol/L). Immediate potassium replacement therapy alleviated symptoms. Further evaluation revealed a new diagnosis of hyperthyroidism, with subsequent treatment initiated (carbimazole and propranolol) preventing recurrence of symptoms. This case highlights the importance of recognizing HPP as a potential manifestation of thyroid dysfunction, particularly in individuals of Asian ethnicity.

## Introduction

Thyrotoxic periodic paralysis (TPP) is a rare and potentially life-threatening manifestation of hyperthyroidism. It predominantly targets young Asian males aged between 20-40 years [1], despite hyperthyroidism being more prevalent among females. This disorder is characterized by abrupt paralytic episodes coupled with lowered potassium levels, occurring concurrently with hyperthyroidism. Achieving a euthyroid state resolves symptoms, emphasizing the importance of diagnosis and definitive treatment. Typically, individuals experience symptoms either in the early morning hours or after rest following strenuous exercise, high carbohydrate meals, or stress [2]. These episodes of paralysis, which can last from hours to days, are typically transient. However, prompt correction of hypokalaemia results in rapid resolution of symptoms [3]. In this context, we present a case of TPP in a 40-year-old Asian male.

## Case presentation

A 40-year-old Asian male presented to the acute medicine unit after experiencing sudden onset proximal limb weakness with generalized body aches and palpitations. These symptoms started a day after returning from a trip to Spain, during which he engaged in heightened physical activity. A similar episode of transient limb weakness had also occurred during the trip. There were no associated respiratory symptoms, recent illnesses, or injuries. The patient's medical history was unremarkable with no significant prior medical conditions or surgeries. He consumed alcohol socially and had no record of smoking or recreational drug use.

Upon examination, we noted reduced power in both lower limbs (1/5) and upper limbs (3/5), as per the Medical Research Council scale for strength. Muscle tone was preserved, and there was lower limb hyporeflexia. There was no goitre with normal neck examination and no thyroid eye disease. There was a mild tremor of the hands. Mobility was reduced and he required a wheelchair during his hospital stay.

**Figure 1:**
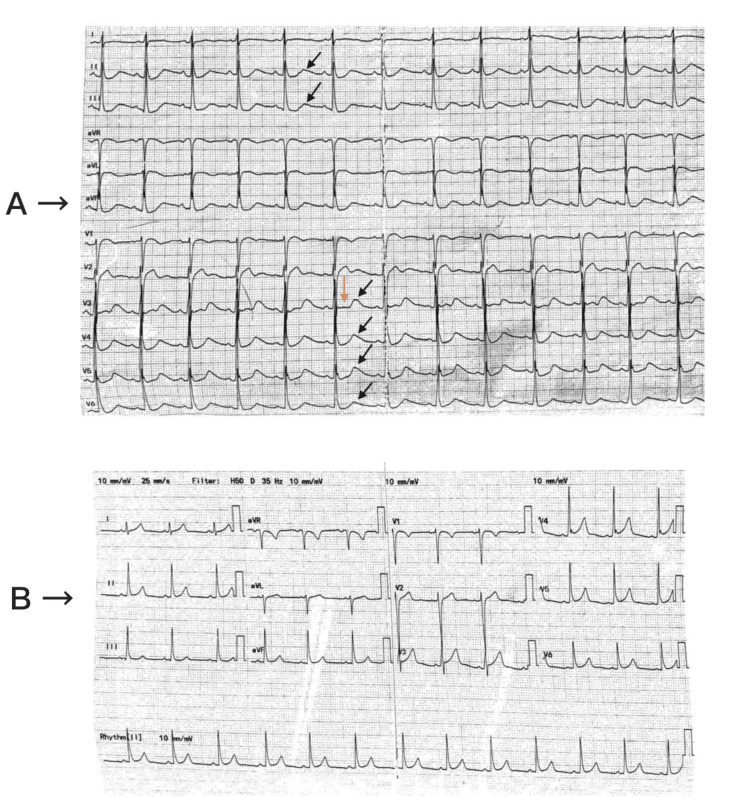
(A) ECG with flat T-waves (orange arrow) and global U-waves (black arrow); (B) Resolution of hypokalemic ECG changes following intravenous potassium infusion.

Laboratory tests revealed a very low serum potassium level of 1.8 mmol/L, while urea and other electrolyte levels, including magnesium, were within the normal range. Full blood count, kidney function, liver function, and C-reactive protein levels were normal. An electrocardiogram (ECG) (Figure 1A) displayed flat T waves and global U waves, but PR and QT intervals were not prolonged. Subsequent investigations showed suppressed levels of thyroid-stimulating hormone (TSH) (<0.05 miu/L) and elevated levels of free thyroxine (free T4) (37.1 pmol/L).

Treatment of severe hypokalaemia was initiated, with the administration of intravenous potassium chloride 0.3% (40 mmol in 1000 ml) over an eight-hour period. This intervention notably improved the symptoms. Consequently, a diagnosis of TPP was established. After the potassium infusion, a repeat blood test indicated a normalized potassium level of 5 mmol/L, and a subsequent ECG showed resolution of previous changes (Figure 1B). The patient was then prescribed carbimazole (40 mg) and propranolol (20 mg, thrice a day) for thyrotoxicosis management. Once his condition stabilized with normal serum potassium levels, he was discharged.

Further follow-up appointments were scheduled to monitor both thyroid function and serum potassium levels, which consistently remained within the normal range. Since discharge, there have been no further episodes of periodic paralysis.

## Discussion

Hypokalaemic periodic paralysis (HPP) is a rare but potentially life-threatening manifestation of various underlying conditions, and it is characterized by episodes of muscle weakness or paralysis associated with low serum potassium levels. While HPP can result from a variety of causes, one of the less common but significant triggers is hyperthyroidism, as illustrated in this case report.

The diagnosis of TPP is supported by the presence of hypokalaemia, elevated levels of free thyroxine, and suppressed TSH during an acute attack [4]. It is more commonly reported in Asian males aged between 20 and 40 years [1].

Hyperthyroidism, an overactive thyroid gland, is more commonly associated with symptoms like weight loss, palpitations, heat intolerance, and tremor. However, it can also lead to TPP with an incidence of 2% [5]. This is characterized by recurrent episodes of muscle weakness or paralysis due to shifts in potassium levels in the body. The proposed mechanism underlying the potassium shifts during the attack can be attributed to the heightened activity of the sodium-potassium pump (Na-K ATPase pump) within skeletal muscles [6]. The heightened pump activity is a result of the direct influence of thyroid hormones, which can boost the genetic transcription of genes responsible for the pump [7]. Additionally, thyroid hormones induce beta-2 adrenergic stimulation, enhancing sensitivity to circulating catecholamines. This heightened sensitivity further augments pump activity. The pump activation is also indirectly triggered by hyperinsulinemia and the influence of androgens. This role of androgens may help elucidate the statistical observation of TPP predominantly affecting males over females, with a ratio of 30:1 [6,8]. As supported by existing literature, individuals typically experience symptoms following a high-carbohydrate meal, which can be explained by the influence of hyperinsulinemia indirectly affecting the sodium-potassium pump. Similarly, symptoms can also manifest after engaging in unaccustomed exercise, as was the case with our patient.

Interestingly, one-third of individuals with TPP have been shown to have mutations that result in the loss of function in genes responsible for muscle-specific inwardly rectifying potassium channels (K_ir_2.6), which can hinder the outward flow of potassium from skeletal muscle cells, causing an imbalance in potassium homeostasis [9].

It is important to consider other potential causes of hypokalaemic periodic paralysis, such as familial periodic paralysis [10], renal tubular acidosis, and medications (e.g., diuretics or laxatives).

The case report highlights the significance of promptly addressing HPP. Low serum potassium levels can lead to muscle weakness, including respiratory muscles, which can result in respiratory complications and even respiratory failure in severe cases. Fortunately, in this case, the patient did not experience respiratory compromise, with prompt treatment initiation. Similarly, hypokalaemic ECG changes if left untreated can progress to severe arrhythmias including sinus arrest, second-degree atrioventricular (AV) block, ventricular tachycardia, and ventricular fibrillation [11].

The intravenous administration of potassium, as performed in this instance, swiftly rectified the potassium imbalance, leading to a notable improvement in the patient's muscle weakness and ECG changes. This intervention not only alleviated his symptoms but also served as a preventive measure against potential complications linked to severe hypokalaemia. Nonetheless, it is imperative to closely monitor potassium replacement to prevent the risk of rebound hyperkalaemia [12]. Achieving an euthyroid state by treating hyperthyroidism helped to prevent the recurrence of future symptomatic episodes.

## Conclusions

HPP, though uncommon, can pose life-threatening risks and often serves as a manifestation of various underlying conditions, such as hyperthyroidism. Effective treatment consists of addressing both the low serum potassium levels and the root cause of HPP, as demonstrated in our case with hyperthyroidism. It is strongly recommended to maintain continuous cardiac monitoring and monitor potassium levels to ensure a safe and successful treatment outcome. Timely diagnosis and treatment are of paramount importance in preventing complications and improving a patient's overall quality of life.
